# Feasibility of online non‐rigid motion correction for high‐resolution supine breast MRI

**DOI:** 10.1002/mrm.29768

**Published:** 2023-06-28

**Authors:** Karyna Isaieva, Camille Meullenet, Pierre‐André Vuissoz, Marc Fauvel, Lena Nohava, Elmar Laistler, Mohamed Aziz Zeroual, Philippe Henrot, Jacques Felblinger, Freddy Odille

**Affiliations:** ^1^ IADI, Université de Lorraine, INSERM U1254 Nancy France; ^2^ Institut de Cancérologie de Lorraine Alexis Vautrin Vandoeuvre‐les‐Nancy France; ^3^ CIC‐IT 1433, INSERM, CHRU de Nancy Nancy France; ^4^ High Field MR Center, Center for Medical Physics and Biomedical Engineering Medical University of Vienna Vienna Austria

**Keywords:** breast MRI, motion correction, online reconstruction

## Abstract

**Purpose:**

Conventional breast MRI is performed in the prone position with a dedicated coil. This allows high‐resolution images without breast motion, but the patient position is inconsistent with that of other breast imaging modalities or interventions. Supine breast MRI may be an interesting alternative, but respiratory motion becomes an issue. Motion correction methods have typically been performed offline, for instance, the corrected images were not directly accessible from the scanner console. In this work, we seek to show the feasibility of a fast, online, motion‐corrected reconstruction integrated into the clinical workflow.

**Methods:**

Fully sampled T_2_‐weighted (T_2_w) and accelerated T_1_‐weighted (T_1_w) breast supine MR images were acquired during free‐breathing and were reconstructed using a non‐rigid motion correction technique (generalized reconstruction by inversion of coupled systems). Online reconstruction was implemented using a dedicated system combining the MR raw data and respiratory signals from an external motion sensor. Reconstruction parameters were optimized on a parallel computing platform, and image quality was assessed by objective metrics and by radiologist scoring.

**Results:**

Online reconstruction time was 2 to 2.5 min. The metrics and the scores related to the motion artifacts significantly improved for both T_2_w and T_1_w sequences. The overall quality of T_2_w images was approaching that of the prone images, whereas the quality of T_1_w images remained significantly lower.

**Conclusion:**

The proposed online algorithm allows a noticeable reduction of motion artifacts and an improvement of the diagnostic quality for supine breast imaging with a clinically acceptable reconstruction time. These findings serve as a starting point for further development aimed at improving the quality of T_1_w images.

## 1 INTRODUCTION

Breast cancer typically remains asymptomatic when the tumor is small; therefore, screening programs are essential for early diagnosis.^1^ MRI is the preferred imaging modality for younger women with dense breast tissues^2^ and is recommended for women presenting high‐risk factors. It enables lesion detection and characterization, clinical staging, and treatment planning. Standard clinical protocols are performed with the patient lying in the prone position (which minimizes respiratory motion in the breast) using a dedicated breast coil array. However, other breast procedures, such as ultrasonic investigations, biopsy, or mastectomy, are usually conducted in the supine position. Being a highly deformable organ, the breast can undergo deformations of 3 to 6 cm between prone and supine positioning.^3^, ^4^, ^5^ This issue is especially critical in the case of tumors invisible by ultrasound^6^ when MRI serves as the principal modality for preoperative tumor localization. Another issue is patient comfort. The examination in the prone position is not well tolerated by some patients (usually, by those who are corpulent and/or suffering from dorsolumbar pain). Moreover, the installation in this configuration becomes an issue for elderly or immobilized patients, and for some specific categories of patients (pregnant women, patients with a colostomy, etc.). Additionally, MRI in the prone position does not allow the application of devices that improve its tolerance by claustrophobic and anxious women (such as immersive video glasses). All of these issues indicate a need to search for an alternative to prone breast MRI.

Performing the examination in the supine position improves the quality of the surgical planning,^7^, ^8^ enables straightforward merging with other modalities (such as echography, CT, or optical surface scanning),^9^ and facilitates biopsy guidance. The exam in the supine position is accessible to a wider range of patients, decreases setup time, does not require complete disrobing of the upper body, and is more comfortable (which minimizes the risk of involuntary bulk motion). However, supine MRI is sensitive to respiratory motion. Multiple strategies have been applied to achieve acceptable image quality with supine MRI. It was shown^10^ that supine breast MRI performed within a breath hold allows tumors to be localized with good precision, however, this strategy cannot be applied to high‐resolution MRI with typical acquisition times of one to several minutes. Some studies have shown that dynamic contrast‐enhanced supine MRI had a diagnostic quality similar to prone MRI if the patient avoided deep breathing.^9^, ^11^ However, a motion correction technique would still be desirable to correct for residual blurring and ghosting. Expiratory gating^12^ and its combination with phase‐encode reordering^13^ allow good quality imaging; however, they increase acquisition time substantially. An alternative approach, suited for MRI‐guided intervention, is prone‐to‐supine lesion mapping. This can be done from a low‐quality supine MRI scan and a high‐quality contrast‐enhanced prone MRI scan^14^ using non‐rigid image registration. However, such an acquisition protocol is complex because it requires imaging in both positions.

Finally, reconstruction frameworks have been developed for free‐breathing MRI with non‐rigid motion correction.^15^ Non‐rigid methods operate with $N_{\dim}\times N_{x}\times N_{y}\times N_{z}$ (number of dimensions multiplied by matrix sizes in all dimensions) motion parameters making this problem extremely ill‐posed. A motion model, linked to a navigator or motion sensors, allows a better determination of the displacement fields. However, the solution to such problems is usually time consuming and requires either sequence reprogramming or external physiological data automatic on‐the‐fly management. Because of these technical challenges, non‐rigid motion correction methods are generally limited to offline use (i.e., the corrected images are not directly accessible from the scanner console). This is a major drawback for the clinical adoption of such methods, as the image quality needs to be inspected quickly to decide whether the acquisition protocol is complete.

The goal of this work is to demonstrate the feasibility of fast, online (i.e., integrated into the regular reconstruction workflow) non‐rigid motion correction for supine breast MRI, practicable in the clinical workflow. The proposed technical solution is based on a recently published hardware and software platform^16^ allowing the integration of external devices, including motion sensors, with a third‐party MRI reconstruction platform. The contribution of the present work is (1) a method to optimize the trade‐off between image quality and reconstruction time (using parallel computing techniques), and (2) an image quality assessment using objective metrics and radiologist scoring, including a comparison with conventional prone images.

## 2 METHODS

### 2.1 Volunteers

The participants were six healthy women 23 to 67 years old (see Table 1). One more volunteer S0 (47 years, 174 cm, 80 kg) was used for a parameter optimization test. Additionally, one volunteer with previously known benign lesions (46 years, 168 cm, 70 kg) was involved in the study for the visual estimation of the diagnostic quality of images. All participants provided written informed consent. The study was conducted under the approved ethical protocol “METHODO” (ClinicalTrials.gov Identifier: NCT02887053) for the healthy volunteers and under the approved ethical protocol “EDEN” (ClinicalTrials.gov Identifier: NCT05218460) for the pathological volunteer, in accordance with the Declaration of Helsinki.

**TABLE 1:** Relevant volunteers' information.

Volunteer code	Age (years)	Height (cm)	Weight (kg)	Breast support
S1	35	163	70	No
S2	23	182	63	Yes
S3	23	158	54	Yes
S4	61	168	66	Yes
S5	54	178	78	Yes
S6	67	160	76	No

Four participants (subjects S2–S5) wore a flexible breast support to allow the reconstruction to be tested with different types of breast deformation patterns.

### 2.2 Data acquisition

The acquisition was performed on a 3T MR scanner (MAGNETOM Prisma, Siemens Healthineers). The protocol included a T_2_‐weighted 2D turbo spin echo (TSE) and a non‐contrast‐enhanced T_1_‐weighted 3D fast low angle shot gradient echo (GRE) sequence. The sequences were launched in two different volunteer positions: prone with a conventional 18‐channel breast coil (Breast 18, Siemens Healthineers) and supine with an 18‐channel multi‐purpose flexible coil above the breasts and a spine coil on the back (Body 18 and Spine Matrix, Siemens Healthineers). It should be noted that the Body 18 coil, being not designed for breast imaging, cannot provide the same SNR of the breast region as the dedicated breast coil within the same measurement time. For this reason, in an average breast volume, an image quality decrease in the supine position is expected.^17^

In the supine position, the arms were placed along the body, and foam spacers were placed between the coil and breast tissue to reduce breast compression. The frequency encoding direction was anterior–posterior, to avoid artifacts from the heart beating.

#### 2.2.1 T_2_w 2D TSE

In the prone position, 80 axial slices with 2 mm thickness were acquired (TR = 5600 ms, TE = 97 ms) in 3 min 22 s with an acceleration factor of 3. In the supine position, 60 axial slices with 3 mm thickness were acquired (TR = 4880 ms, TE = 102 ms) in 6 min 21 s. Disabled parallel imaging (therefore, longer acquisition), together with a larger slice thickness, allowed for achieving a comparable SNR in the supine position (using a non‐dedicated coil) and a better motion model quality. Because the T_2_w 2D sequence is not used for dynamic contrast enhancement imaging, a longer acquisition would still be acceptable for clinical applications. Saturation bands were added on the arms, for supine position MRI, to avoid their potential aliasing into the breast FOV. The in‐plane resolution was 0.72 mm in a FOV of 402 mm × 230 mm.

#### 2.2.2 T_1_w 3D GRE

For the T_1_ 3D GRE sequence, we kept the same acquisition protocol for the prone and the supine positions. The temporal resolution was 2 min 15 s per 3D volume. Minor differences were caused by different geometry in these two positions. TE was 1.98 ms for the prone position and 1.93 ms in the supine position. TR was 4.14 ms in both cases. The images were acquired with an acceleration factor of 2 in the in‐plane phase encoding direction. A total of 192 axial images with a slice thickness of 1 mm were reconstructed. The in‐plane resolution was 0.75 mm in the case of the prone position and 0.79 for the supine position. The FOV was 367 × 240 mm in the prone position and varied from 387 × 252 mm to 459 × 300 mm in the supine position.

#### 2.2.3 Motion measurements

A pneumatic respiratory belt, connected to a modified patient monitoring system (Maglife, Schiller Médical) was placed on the patient's chest (just below the breast) to measure their respiratory activity in the supine position. The belt is made of a corrugated tube that extends during the inspiration and contracts during the expiration. The tube is hermetically connected to a pressure sensor with rubber tubing. Therefore, the sensor's indications reflect the respiratory position. The respiratory belt signals were automatically recorded by the signal analyzer and event controller system.^16^

Additionally, a real‐time radial FLASH sequence^18^ (TR = 2.22 ms, TE = 1.46 ms, in‐plane resolution = 1.6 mm, slice thickness = 10 mm, 30 frames/s) was run on one breast slice, for direct visualization of breast motion during free breathing.

### 2.3 Image reconstruction

#### 2.3.1 Joint reconstruction of image and motion

Motion‐correcting reconstruction was done with the generalized reconstruction by inversion of coupled systems (GRICS) method.^15^, ^19^ This algorithm solves for both a motion‐corrected image *ρ*_*0*_ and the parameters of a motion model *α*, that links respiratory belt signals s(t) (acquired simultaneously with the MRI sequence) with actual in‐plane motion u(x,y,t) (displacement of each image pixel). The problem can be formulated as follows:

$$\left\{\begin{array}{ll} \rho_{0},\alpha =\underset{\rho_{0},\alpha }{\arg\min}{\left\|E(u)\rho_{0}-m\right\|}^{2}+\lambda {\left\|\rho_{0}\right\|}^{2}+\mu \|\nabla \alpha {\|}^{2}, & \left(1a\right)\\ u(x,y,t)=\alpha (x,y)s(t). & \left(1b\right) \end{array}\right.$$

Here, *m* is the acquired MRI k‐space data, *E* is an encoding operator (which is comprised of k‐space sampling operators, Fourier transformations, coil sensitivity weightings and spatial transformation operators depending on u[α(x,y),s(t)]), u are the displacement fields, and s is the sensor (respiratory belt) signal. A Tikhonov regularization was applied to each equation with regularization parameters *λ* = 1 for the generalized reconstruction and *μ* = 0.5 for the model optimization. The coil sensitivity maps were calculated from the central lines of k‐space data (32 lines in case of 2D TSE and 24 calibration lines in case of 3D GRE) and smoothed with splines with smoothing coefficient *λ*_smooth_ = 1000 (for both magnitude and phase).

The optimization was done by performing these reconstruction and motion model steps on different scales starting from the lowest resolution, iteratively going to the native image resolution with a number of iterations $N_{\text{iter}}$ (fixed‐point iterations). Each iteration of these two steps was solved with an iterative conjugate gradient solver with a maximum number of iterations ${\text{Maxit}}_{\text{recon}}$ and ${\text{Maxit}}_{\text{motion}}$ and tolerance of 10^−3^ for the reconstruction solver and to 10^−2^ for the model solver, respectively. In the final resolution level, only the reconstruction step was performed. The respiratory sensor signal was re‐binned to $N_{s}$ motion states.

The respiratory signal was filtered with a low‐pass filter with a cutoff frequency of 3 Hz. A respiration curve drift (which is usually caused by air leaks) correction (modeled with a quadratic function) was also applied. The resulting curve was obtained by subtraction of the fitted drift function.

#### 2.3.2 Implementation for online use

The image reconstruction in the supine position was performed online within the Gadgetron framework.^20^ For physiological data reception, the reconstruction server communicated with the signal analyzer and event controller system as described in.^16^ Online and offline reconstruction time was calculated as the sum of the time extracted from Gadgetron logs (between the reception of the last k‐space line and the end of the reconstruction) and a transfer delay, which was set to 2 s.^16^

##### T_2_w 2D TSE

The reconstruction (online and retrospective simulations) was performed on a workstation with 48 cores (96 threads) with 3.7 GHz and 256 GB RAM. Here and below, by “retrospectively,” we mean “manually launched after the sequence end,” contrary to “online,” which means “without any intervention and with automatic images reception on the console.” GRICS reconstruction and coil sensitivity maps estimation of each slice were run by an individual thread (using standard C++ threads). The manufacturer's reconstruction for the supine position was performed retrospectively using Siemens XBuilder tool on the MRI console right after the acquisition.

##### T_1_w 3D GRE

**FIGURE 1:**
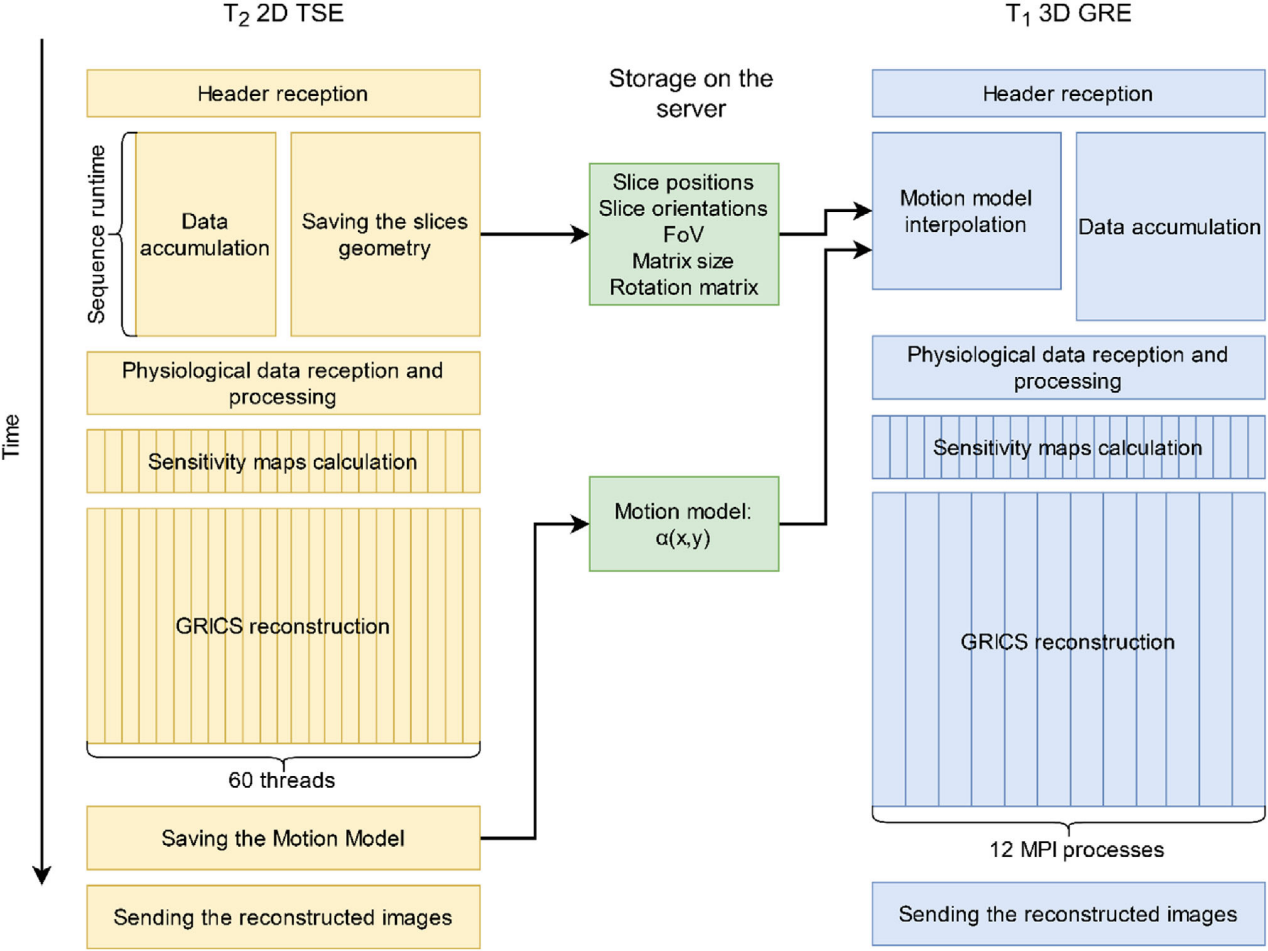
Schematic illustration of the reconstruction pipelines for T_2_‐weighted (T_2_w) and T_1_‐weighted (T_1_w) sequences.

The reconstruction was performed retrospectively using the manufacturer's reconstruction environment (Siemens ICE simulator) on a workstation with 64 cores (128 threads) of 4 GHz and 1024 GB RAM. The 3D GRICS reconstruction was parallelized in the following way: 12 Message Passing Interface (MPI) processes were launched, each of which worked with different motion states. Each of the MPI processes disposed of maximum of 10 OpenMP threads, which performed the calculations each for a different coil. Considering T_1_w 3D GRE sequence was launched after T_2_w 2D TSE, the motion optimization step was omitted, and the motion model was obtained from the previous reconstruction (which was launched beforehand on the server) by interpolation of the α maps used to calculate the motion field according to Eq. (1b) (see Figure 1). A separate test was performed on a phantom to ensure that the motion model interpolation was performed during the sequence runtime. The manufacturer's reconstruction for the supine position was obtained in a conventional way during the exam.

For the pathological volunteer, the motion‐corrected reconstruction was performed online and the non‐corrected image was obtained retrospectively using the Siemens built‐in tool on the scanner console.

#### 2.3.3 Hyperparameter optimization

##### T_2_w 2D TSE

We aimed at optimizing the main reconstruction parameters that affect image quality and reconstruction time. We varied the parameters described hereafter to find an optimal trade‐off between image quality and reconstruction time per slice. The number of fixed‐point iterations $N_{\text{iter}}$ was varied from 3 to 4; the number of motion states $N_{s}$ from 6 to 16 with steps of 2; the maximal number of iterations for both solvers was varied from 5 to 20 with a step of 5. Given a smooth character of the motion correction improvement with an increase of these parameters, we selected the parameter set, which gave the highest sharpness index^21^ under the constraint that the reconstruction time was <90 s. The test was performed offline for subject S0, using Siemens ICE simulator, and the selected parameters were then used for the online reconstruction for subjects S1–S6.

##### T_1_w 3D GRE

The optimization was performed fully retrospectively on the full set S1–S6 using Siemens ICE simulator. The number of motion states varied from 4 to 16 with the step 4 (the number of MPI processes). The maximal number of iterations for the reconstruction solver was set to 10. The sharpness index (normalized by that of the non‐corrected image), was selected as the quality metric for this test. The motion was considered corrected in case of saturation of the sharpness index with the number of motion states increasing. The number of motion states was therefore, selected manually, by analyzing the sharpness index curves, and confirmed by visual inspection of reconstructed images. The final number of motion states was selected so that the motion artifacts were corrected for all subjects.

#### 2.3.4 Postprocessing

The images were normalized by pixelwise division by the sum of squares of the coil sensitivities (online implementation).

To minimize the impact of residual motion and aliasing artifacts (manifesting outside of the breast) on quantitative metrics, the images were automatically segmented with a k‐means‐based segmentation algorithm, and the background was masked out (offline implementation for quantitative evaluation only). The detailed algorithm used in the case of T_2_w 2D TSE images is provided in File S1. The resulting images were visually evaluated to ensure segmentation correctness. The T_1_w 3D GRE images were segmented manually in the ITK Snap software.^22^

Additionally, the plausibility of the GRICS motion model (α) was verified by applying the motion model to the real‐time FLASH sequence. The motion‐corrected T_2_w images were warped using the motion fields predicted from $u_{\text{real}‐\text{time}}\left(x,y,t\right)=\alpha \left(x,y\right)s_{\text{real}‐\text{time}}\left(t\right)$, for direct comparison with the real‐time, dynamic breast images during free breathing.

Similarly, the motion model was applied to the 3D surface of the breast, which was obtained by adaptive thresholding of the T_2_w 2D TSE images. A segment of 10 s was taken from the respiratory data acquired during the T_2_w sequence to illustrate the chest motion. The motion direction was calculated from the difference between the displacement of the current and previous frames $\mathrm{du}\left(x,y,t_{i}\right)=u\left(x,y,t_{i}\right)-u\left(x,y,t_{i-1}\right)$. A moving average filter with a window size of three was applied in the *z*‐direction.

### 2.4 Quality assessment

#### 2.4.1 Motion model evaluation

The predicted breast motion (obtained as described above in the Postprocessing subsection) was qualitatively compared to the actual motion acquired with the real‐time radial FLASH sequence by their simultaneous visualization.

#### 2.4.2 Quantitative evaluation by objective metrics

For each slice acquired in the supine position (6×60 for T_2_w images and 6×192 T_1_w images), image quality was estimated using two metrics: sharpness index (SI) (definition 4 from Leclaire and Moisan)^21^ and average edge strength (AES) (equation 4 from Aksoy et al. and Zacà et al.)^23^, ^24^ Sharpness enhancement was defined as $\mathrm{SE}=2\left(SI_{\text{GRICS}}-SI_{\text{uncorr}}\right)/\left(SI_{\text{GRICS}}+SI_{\text{uncorr}}\right)$, where $SI_{\text{GRICS}}$ is the sharpness index of an motion‐corrected image and $SI_{\text{uncorr}}$ is the sharpness index of an image reconstructed with the manufacturer's uncorrected reconstruction. Similarly, average edge enhancement was calculated as $\mathrm{AEE}=2\left({\mathrm{AES}}_{\text{GRICS}}-{\mathrm{AES}}_{\text{uncorr}}\right)/\left({\mathrm{AES}}_{\text{GRICS}}+{\mathrm{AES}}_{\text{uncorr}}\right)$.

To estimate if motion correction improved quality (in terms of the calculated metrics), a paired Student t‐test was performed with *p* < 0.05 being considered statistically significant. A Kolmogorov–Smirnov test was performed to ensure that the quantities were normally distributed (*p* > 0.05 was considered normal).

#### 2.4.3 Visual evaluation by radiologists

The visual evaluation was performed by two radiologists: R1 (C.M., 2 years of experience) and R2 (P.H., 25 years of experience). The images were fully randomized so that subject identification, acquisition, and reconstruction type were unknown to the radiologists. A radiological score (from 1 to 5) was given to strength of motion artifacts, uniformity, and overall quality of the whole multi‐slice dataset, respectively.

Weighted Cohen's κ was calculated to verify the consistency between the scores of the two radiologists. κ > 0.6 was considered substantial.^25^ A Wilcoxon signed‐rank test was used to compare the quality: (1) between uncorrected and corrected supine images, and (2) between prone images and GRICS supine images. A *p*‐value <0.05 was considered statistically significant. All statistical tests were performed in R.^26^

## 3 RESULTS

### 3.1 Reconstruction parameter trade‐off

#### 3.1.1 T_2_w 2D TSE

**FIGURE 2:**
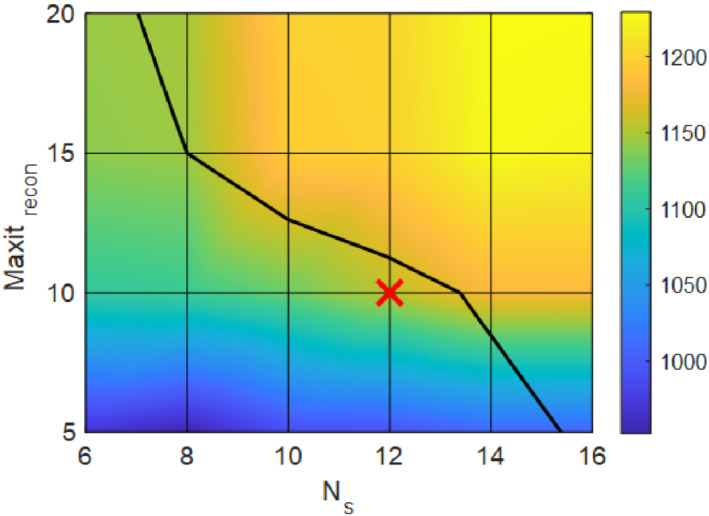
Dependence of average sharpness index of two central slices of the volunteer S1 (encoded with color) on the number of motion states $N_{s}$ and maximal number of iterations of the reconstruction solver ${\text{Maxit}}_{\text{recon}}$. The grid intersections represent the actually calculated values and the color plot was interpolated. The black line is the isoline corresponding to a reconstruction time of 90 s (the top right part of the plot had reconstruction time >90 s). The red cross points to the selected set of reconstruction parameters.

It was found that the sharpness index and reconstruction time were not much affected by the number of fixed‐point iterations $N_{\text{iter}}$, and the maximal number of iterations of the motion solver. They were chosen to be as follows: $N_{\text{iter}}$ = 4, ${\text{Maxit}}_{\text{motion}}=10$. However, the number of motion states $N_{s}$ and the maximal number of iterations for the reconstruction solver ${\text{Maxit}}_{\text{recon}}$ affected the sharpness index and reconstruction time as depicted in Figure 2. Given the 90 s constraint, the reconstruction parameter optimum was found at $N_{s}=12$, ${\text{Maxit}}_{\text{recon}}=10$.

One can also note that even higher values of $N_{s}$ and ${\text{Maxit}}_{\text{recon}}$ do not lead to further substantial quality improvement (sharpness index saturates). For comparison, the sharpness index of two central slices of subject S1 was: 478 without motion correction, 1155 for the selected motion correction parameter set (81 s reconstruction time), and 1245 when the convergence at the selected tolerance was reached (after 314 s).

The total online reconstruction time was 110 to 118 s.

#### 3.1.2 T_1_w 3D GRE

**FIGURE 3:**
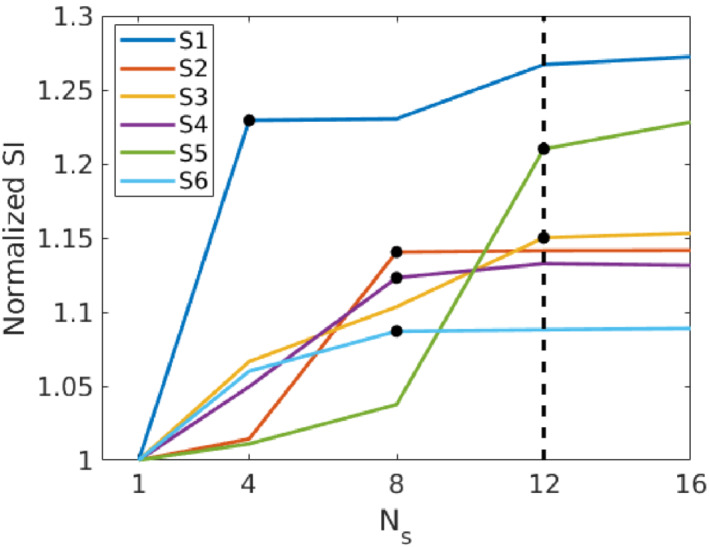
Dependence of normalized sharpness index on number of motion states. Black dots represent manually selected number of motion states where the sharpness index saturates. The dashed vertical line represents the finally selected value.

It was found that the calculation time grew approximately linearly with the number of motion states with the slope of (0.65±0.07)×(calulation time for4motion states). The normalized sharpness index dependency on the number of motion states is shown in Figure 3. The manually selected threshold values of the number of motion states were confirmed by direct visual evaluation of the reconstructed images. The selected final number of motion states was $N_{s}=12$.

The pure time of the GRICS reconstruction was 72 to 105 s, and the total reconstruction time after the supposed end of the sequence was 109 to 157 s (with an average reconstruction time of 130 s).

### 3.2 Motion model verification and visualization

The comparison between the warped T_2_w images (using the GRICS motion model) and the real‐time FLASH sequence is shown in Video S1. The video shows a good agreement between the breast motion synthesized by the GRICS motion model and the actual real‐time breast images.

The 3D surface motion visualization is presented in Videos [Link], [Link], [Link], [Link], [Link], [Link]. One can observe that the in‐plane motion patterns were quite similar in the case of no breast holder (S1 and S6), whereas the volunteers who wore the holder (S2–S5) demonstrated diverse motion patterns: from almost vertical motion (S2) to a complex deformation pattern, which was probably caused by the breast compression (S5).

### 3.3 Quantitative evaluation

Quantitative metrics are summarized in Table 2.

**TABLE 2:** Quantitative metrics summary: SI, SE, AES, and AEE.

	T_2_w 2D TSE						T_1_w 3D GRE					
Subject	SI_std_, mean (std)	SI_GRICS_, mean (std)	SE, %	AES_std_, mean (std)	AES_GRICS_, mean (std)	AEE, %	SI_std_, mean (std)	SI_GRICS_, mean (std)	SE, %	AES_std_, mean (std)	AES_GRICS_, mean (std)	AEE, %
S1	731 (248)	1635 (658)	76	1.18 (0.16)	1.49 (0.14)	24	3714 (813)	4060 (1176)	7	1.19 (0.13)	1.22 (0.14)	4
S2	568 (212)	1436 (464)	86	1.36 (0.15)	1.68 (0.19)	20	3256 (690)	4172 (1263)	21	1.06 (0.15)	1.20 (0.10)	14
S3	796 (215)	2134 (894)	92	1.53 (0.33)	1.89 (0.35)	22	2988 (891)	4126 (1340)	30	1.11 (0.17)	1.26 (0.14)	13
S4	470 (208)	1491 (411)	106	1.01 (0.25)	1.39 (0.22)	33	3359 (1313)	4517 (1469)	33	1.03 (0.34)	1.12 (0.34)	9
S5	1166 (268)	3173 (819)	91	1.22 (0.27)	1.48 (0.25)	20	3903 (893)	4682 (911)	19	1.04 (0.12)	1.08 (0.16)	4
S6	797 (248)	1956 (623)	82	1.04 (0.28)	1.31 (0.20)	25	3485 (596)	4048 (1187)	11	0.93 (0.28)	0.98 (0.31)	6

Abbreviations: AEE, average strength enhancement; AES, average edge strength; GRE, gradient echo; GRICS, generalized reconstruction by inversion of coupled systems; SE, sharpness enhancement; SI, sharpness index; std (subscript) standard method; (std), standard deviation; T_1_w, T_1_‐weighted; T_2_w, T_2_‐weighted.

#### 3.3.1 T_2_w 2D TSE

Statistical analysis showed that the difference between the quantitative metrics of corrected and uncorrected images was highly significant (*p* < 0.0001) for both SI and AES. Quality metrics were always higher for the corrected images and average enhancement was 89% for SI and 24% for AES.

#### 3.3.2 T_1_w 3D GRE

Quality metrics were systematically higher for the corrected images on the central slices and could be either higher or slightly lower on the peripherical slices. Nevertheless, the difference between the quantitative metrics of corrected and uncorrected images was highly significant (*p* < 0.0001) for both SI and AES. The average enhancement was 20% for SI and 8% for AES.

### 3.4 Visual evaluation by radiologists

#### 3.4.1 T_2_w 2D TSE

**FIGURE 4:**
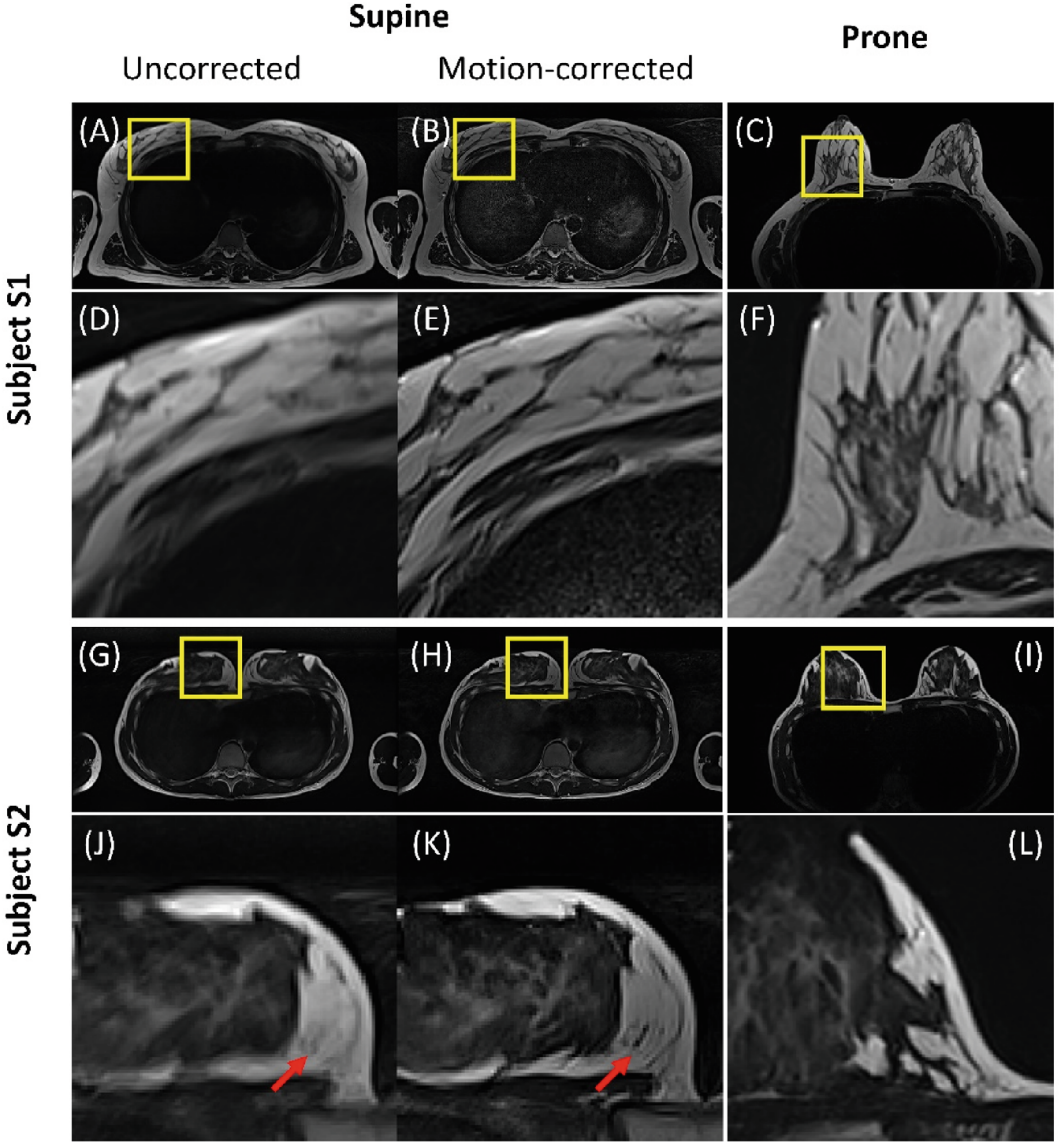
Examples of non‐corrected (A,G) and motion‐corrected (B,H) reconstruction of the T_2_‐weighted 2D TSE sequence illustrated on supine images compared to prone images (C,I) of subjects S1 and S2, respectively. (D–F) and (J–L) show zoomed regions from (A–C) and (G–I). Red arrows point to anatomical features, which are poorly or not visible before correction, and clearly distinguishable after correction.

Direct visual evaluation of two different supine image reconstructions showed that the motion blurring present in the uncorrected images was noticeably reduced by the motion correction. This enabled observation of fine details (e.g., of milk gland structures not visible in uncorrected images). Examples of images with and without correction are provided in Figure 4.

The radiologists' scores are presented in Table 3. The inter‐observer agreement was substantial for sharpness (κ = 0.79) and overall quality (κ = 0.81), and moderate for uniformity (κ = 0.56).

**TABLE 3:** Radiologists' scores for the T_2_w 2D TSE images.

	Motion artifacts						Uniformity						Overall quality					
	Sup. convent.		Sup. GRICS		Prone		Sup. convent.		Sup. GRICS		Prone		Sup. convent.		Sup. GRICS		Prone	
	R1	R2	R1	R2	R1	R2	R1	R2	R1	R2	R1	R2	R1	R2	R1	R2	R1	R2
S1	3	3	4	4	4	5	3	3	5	4	5	4	3	3	4	5	5	5
S2	2	2	4	4	5	5	5	3	5	5	5	5	2	2	4	4	5	5
S3	2	2	4	4	4	4	4	3	4	4	5	4	2	2	4	4	5	4
S4	2	2	3	3	5	5	3	3	5	5	4	4	2	2	3	3	5	5
S5	3	4	4	5	5	5	4	4	5	5	3	3	3	4	5	5	5	5
S6	3	4	4	4	4	4	4	4	5	5	4	4	3	4	4	4	4	4

*Note*: The following criteria were defined: 1. Strength of motion artifacts (1 = “major artifact making the examination uninterpretable” 2 = “major artifact degrading interpretation” 3 = “significant artifact allowing interpretation” 4 = “minor artifact” 5 = “no artifact”); 2. Uniformity (1 = “major signal inhomogeneity making the examination uninterpretable” 2 = “major signal inhomogeneity degrading interpretation” 3 = “signal inhomogeneity allowing interpretation” 4 = “minor inhomogeneity” 5 = “no inhomogeneity”); 3. Overall quality (1 = “very low” 2 = “low” 3 = “average” 4 = “good” 5 = “excellent”). R1 is the junior radiologist, R2 is the senior radiologist.

Abbreviations: Convernt., conventional; GRICS, generalized reconstruction by inversion of coupled systems; sup., supine; T_2_w, T_2_‐weighted; TSE, turbo‐spin‐echo.

The radiologists' scores of GRICS reconstructions were always greater than or equal to those of the uncorrected supine images. The median difference was 1.25 for motion artifacts, 1.16 for uniformity, and 1.42 for overall quality. The statistical analysis has shown that GRICS reconstruction was significantly better in terms of all three investigated parameters: motion artifacts (*p* = 0.034), uniformity (*p* = 0.031), and overall quality (*p* = 0.035).

Comparison of the quality between the GRICS supine images and the prone images did not show significant differences (*p* = 0.097 for motion artifacts, *p* = 0.17 for uniformity, and *p* = 0.097 for overall quality). However, for motion artifacts and overall quality, the radiologists' scores of the prone images were always greater than or equal to those of the GRICS images with a mean difference, 0.67 (for both motion artifacts and overall quality). Uniformity was better for the GRICS images with a mean difference, −0.58.

#### 3.4.2 T_1_w 3D GRE

**FIGURE 5:**
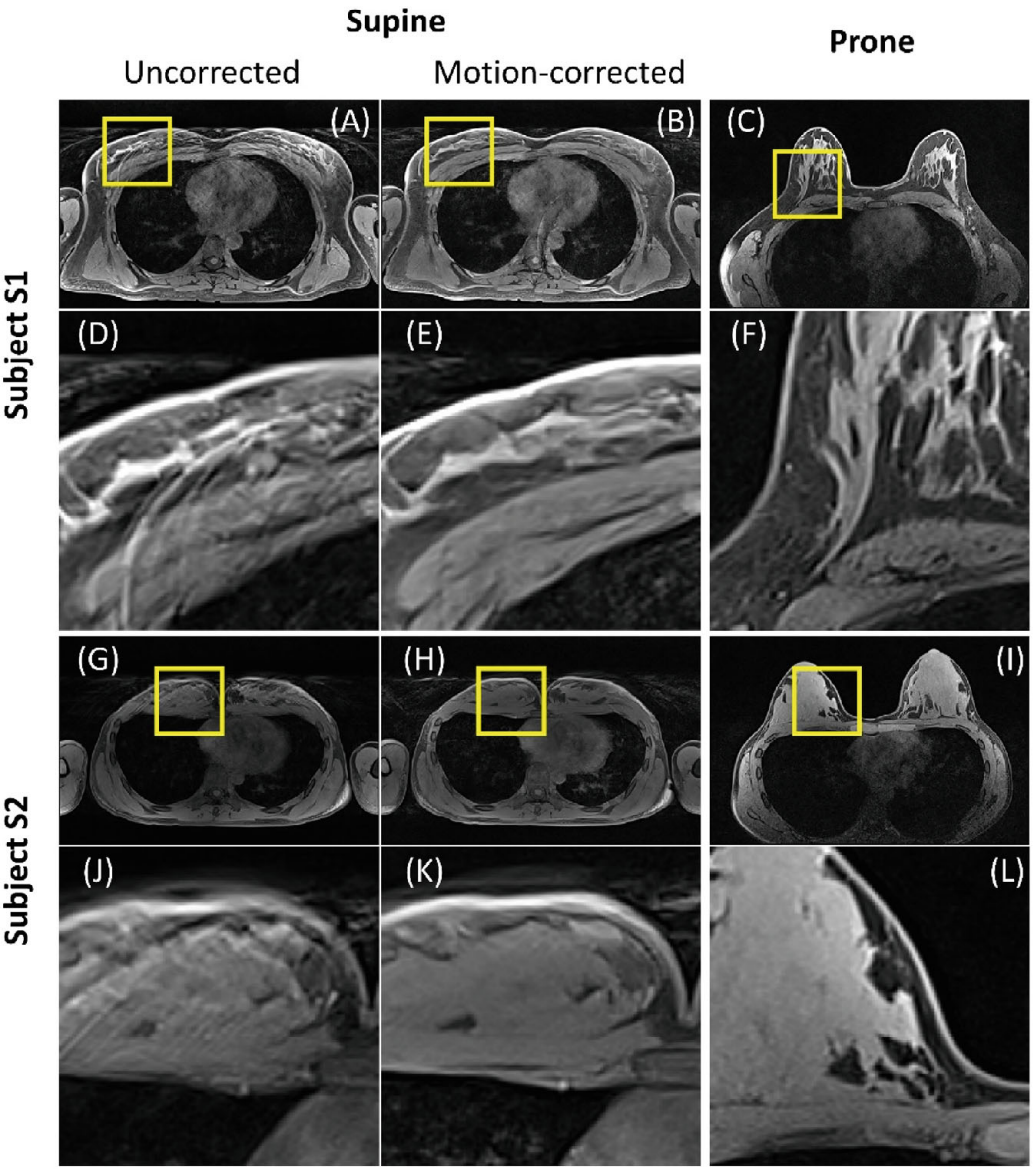
Examples of non‐corrected (A,G) and motion‐corrected (B,H) reconstruction of the T_1_w 3D gradient echo sequence illustrated on supine images compared to prone images (C,I) of subjects S1 and S2, respectively. (D–F) and (J–L) show zoomed regions from (A–C) and (G–I).

Contrary to the T2w 2D TSE sequence, where the motion artifacts led to uniform image blurring, in the case of T_1_w 3D GRE, they manifested in the form of ghosting and anisotropic blur (as can be seen in Figure 5). Visual comparison of the images before and after the correction allowed the conclusion that the majority of artifacts were successfully removed, and only residual artifacts (manifesting more in the peripherical slices) were observed. However, the supine images demonstrated a substantially lower SNR than that of the prone images. For some subjects, residual parallel imaging artifacts were observed on both supine images, manifesting slightly more on the GRICS images.

The radiologists' scores from the randomized blind evaluation for the T_1_w 3D TSE sequence are presented in Table 4. The inter‐observer agreement was high for motion artifacts (κ = 0.72) and overall quality (κ = 0.76), and moderate for uniformity (κ = 0.56).

**TABLE 4:** Radiologists' scores for the T_1_w 3D GRE images.

	Motion artifacts						Uniformity						Overall quality					
	Sup. convent.		Sup. GRICS		Prone		Sup. convent.		Sup. GRICS		Prone		Sup. convent.		Sup. GRICS		Prone	
	R1	R2	R1	R2	R1	R2	R1	R2	R1	R2	R1	R2	R1	R2	R1	R2	R1	R2
S1	2	2	3	3	5	5	4	3	3	3	5	4	2	1	3	2	5	5
S2	2	2	3	3	5	5	4	4	4	4	5	5	2	2	3	3	5	5
S3	2	2	3	3	5	5	3	3	4	4	5	5	2	2	3	2	5	5
S4	1	2	2	3	5	5	3	3	4	3	4	4	1	2	2	2	4	4
S5	2	2	2	3	5	5	3	4	3	4	4	4	2	2	2	2	4	4
S6	2	3	2	3	5	5	2	3	2	2	3	4	3	3	3	2	4	5

*Note*: The following criteria were defined: 1. Strength of motion artifacts (1 = “major artifact making the examination uninterpretable” 2 = “major artifact degrading interpretation” 3 = “significant artifact allowing interpretation” 4 = “minor artifact” 5 = “no artifact”); 2. Uniformity (1 = “major signal inhomogeneity making the examination uninterpretable” 2 = “major signal inhomogeneity degrading interpretation” 3 = “signal inhomogeneity allowing interpretation” 4 = “minor inhomogeneity” 5 = “no inhomogeneity”); 3. Overall quality (1 = “very low” 2 = “low” 3 = “average” 4 = “good” 5 = “excellent”). R1 is the junior radiologist, R2 is the senior radiologist.

Abbreviations: Convent., conventional; GRE, gradient echo; GRICS, generalized reconstruction by inversion of coupled systems; sup., supine; T_1_w, T_1_‐weighted;

Comparison between the GRICS and the uncorrected images confirmed that the strength of motion artifacts significantly decreased with a mean difference of 0.75 and *p* = 0.048. Uniformity did not change (mean difference, 0.08 and *p* = 0.85). Overall quality was better for the GRICS images on average (mean difference, 0.42), however not statistically significant (*p* = 0.17).

Prone images demonstrated significantly higher quality for all three parameters (mean difference, 2.25 and *p* = 0.032 for motion artifacts; mean difference, 1 and *p* = 0.035 for uniformity; and mean difference, 2.17 and *p* = 0.031 for overall quality).

#### 3.4.3 Diagnostic quality

**FIGURE 6:**
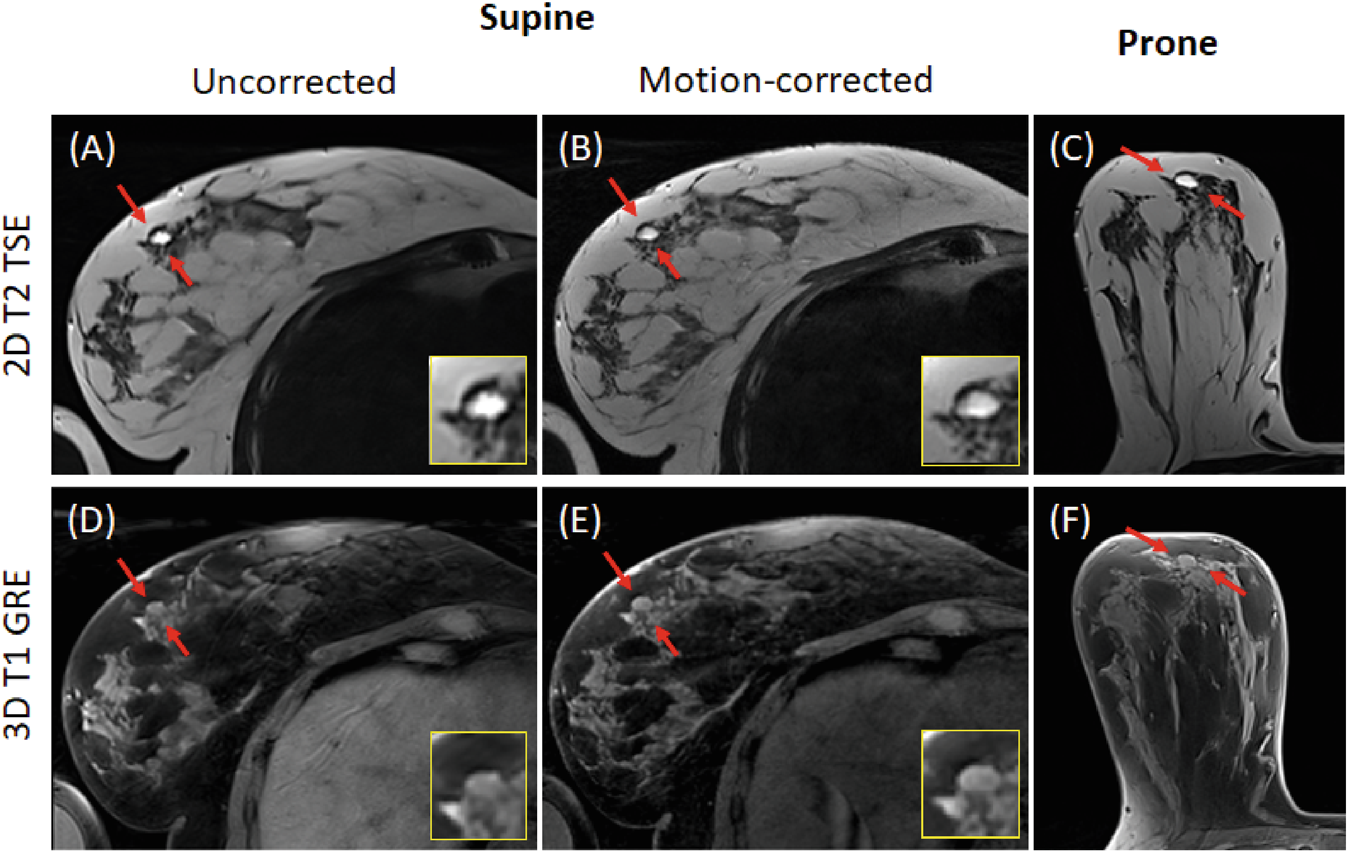
Images zoomed on one breast with the greatest lesion of the pathological volunteer. (A) and (B) illustrate the uncorrected and the motion‐corrected supine T_2_‐weighted (T_2_w) images, (C) demonstrates the prone T_2_w image, (D) and (E) show the uncorrected and the motion‐corrected T_1_‐weighted (T_1_w) images, and (F) illustrated the prone T_1_w image. The red arrows indicate the lesion, and the insets show the zoomed lesion regions. Note that different images have slightly different slice position and thickness, which leads to some differences in the lesion shape.

The benign lesions hypersignal on T_2_w images and hyposignal on T_1_w images allowed a conclusion that these are probably cysts (which usually demonstrate regular boundaries). Figure 6 presents the largest lesion of the pathological volunteer. Although the uncorrected T_2_w images demonstrate irregular lesion shape (which might potentially lead to an erroneous diagnosis as irregular boundaries are a pejorative feature in opposition to regular shape), one can observe regular lesion boundaries on the prone and the motion‐corrected T_2_w images. The non‐corrected T_1_w images do not allow clear lesion shape resolution, and the motion‐corrected T_1_w supine images provide a straightforward oval‐shaped lesion distinction. The diagnostic quality of both acquired T_2_w and T_1_w motion‐corrected supine images was estimated as similar to that of the prone images.

## 4 DISCUSSION

We have shown the feasibility of online implementation of the GRICS algorithm for supine breast MRI. It was demonstrated that, despite the constraint of the GRICS reconstruction time, its application leads to a significant decrease in motion artifacts and improves diagnostic quality. Furthermore, we have shown that full online reconstruction time after the end of the sequence (including motion model interpolation, coil sensitivity maps calculation, etc.) did not exceed 2 min for the T_2_w sequence, and 2 min 40 s for the T_1_w sequence.

Both evaluated quality metrics demonstrated highly statistically significant differences between corrected and uncorrected images. However, such metrics may be affected by multiple factors, such as different normalizations or different coil sensitivities. In the case of T_1_w 3D GRE images, the artifacts manifesting in the form of ghosts, and not only blur, could artificially increase the sharpness while decreasing the image quality. These results, therefore, required confirmation by a blinded visual evaluation by experts in breast radiology. They scored the corrected images significantly lower for motion artifact strength, supporting the reliability of our results. All corrected images of volunteers below 50 years (who have denser breasts and are the target group of the breast MRI) were scored as allowing interpretation by both radiologists.

The comparison of quality metrics of conventional prone and GRICS‐reconstructed supine images was not possible because of the different geometry and contrast level of the images. Visual evaluation by the radiologists showed differences in the strength of motion artifacts and uniformity between conventional prone and motion‐corrected supine images. The former could be caused by incomplete motion correction. The motion model could be improved by increasing the number of motion sensors, which would provide more information on complex non‐rigid chest motion. Adding a second respiratory belt could be useful, however, is not always possible because of the breast anatomy. A preliminary test^16^ demonstrated that accelerometer‐based motion sensors^27^ can also allow for motion correction. In that case, multiple sensors could be easily installed and could potentially improve the motion correction quality.

The overall quality parameter additionally depended on the level of distortion by various artifacts and SNR, and therefore, depended on the coil geometry. It was shown earlier that flexible coils can improve SNR^28^ and potentially also comfort. Considering that the multi‐purpose coil used in this study is suboptimal for breast imaging, moving toward a flexible coil designed specifically for supine breast MRI could improve the SNR by a factor of up to 3^17^ and, therefore, lead to higher overall quality and/or decreased acquisition time.

The proposed method unlocks new opportunities in supine breast imaging, which could serve as an alternative to conventional prone MRI. However, the method has some limitations. Although being close to clinically acceptable, the total time required for the T_1_w sequence (with reconstruction‐related waiting time between the sequences) exceeded the current recommendations for dynamic contrast‐enhanced imaging.^29^ Nonetheless, with the mentioned above hardware optimization and with further software optimization, it may be possible to achieve a satisfactory temporal resolution.

Another constraint of the proposed method is that it corrects for 2D motion occurring in the axial plane, where the most significant displacement typically arises during respiration. However, depending on the patient, the through‐plane motion can occur and degrade the image quality. Despite through‐plane motion correction not being possible for 2D multi‐slice sequences, this issue should not lead to a significant decrease in quality. It may still cause nearly uniform signal drops, but these are not pronounced with relatively slow respiratory breast motion. However, for 3D sequences additional blurring and ghosting in the through‐plane direction is possible. Therefore, for the T_1_w 3D GRE sequence, a 3D motion model might improve the quality of the motion correction. This would require an adjustment of the acquisition protocol. One can imagine a fully sampled T_1_w sequence (allowing a reliable 3D motion model calculation) followed by a series of accelerated contrast‐enhanced T_1_w sequences. Moreover, the addition of this sequence could help in reaching a better precision of coil sensitivity maps, which could reduce parallel imaging artifacts. Therefore, the increased total duration of the protocol (compared to that of the conventional prone protocol) is a limitation that may not be overcome; however, it should still be well tolerated by patients because of its superior physical and psychological comfort.

## 5 CONCLUSION

We demonstrated the feasibility of fast, online, non‐rigid motion correction based on a respiratory belt signal for supine breast MRI, for the principal sequences used in clinical practice. Optimizing the reconstruction parameters and using parallel computing resulted in a total reconstruction time below 2 min for the T_2_w sequence, and below 3 min for the T_1_w sequence.

The efficiency of GRICS motion correction was quantified for supine breast MRI. The resulting images had significantly higher quality metrics (sharpness index and average edge strength) and significantly better radiological scores for motion artifact strength. The motion‐corrected images from the involved pathological volunteer demonstrated a diagnostic quality superior to that of the non‐corrected images and comparable to that of the prone images. For the fully sampled T_2_w 2D TSE GRICS images, radiological scores for uniformity and overall quality were also significantly higher, bringing their quality closer to that of conventional prone images. The accelerated T_1_w GRICS images demonstrated a substantially lower SNR and presence of residual blur (and, therefore, significantly lower overall quality) compared to the prone images, which could be improved in the future with the dedicated hardware and a 3D motion model.

## FUNDING INFORMATION

Joint French‐Austrian project “BraCoil”, Grant/Award Number: ANR‐17‐CE19‐0022, Agence Nationale de la Recherch France, and I3618‐B33, Austrian Science Fund FWF, Austria; France Life Imaging; CPER IT2MP and FEDER.

## CONFLICT OF INTEREST STATEMENT

J.F. and F.O. are co‐founders and share‐holders of Epsidy (Nancy, France).

## Supporting information


**File S1.** Background masking algorithm.

**Video S1.** Comparison of motion of a warped GRICS‐reconstructed image (on the top left) and actual motion recorded with a real‐time FLASH sequence (on the top right). The GRICS images were obtained by applying the motion fields predicted from GRICS motion parameters and the respiration data recorder during the real‐time MRI acquisition (shown on the bottom).

**Video S2.** The breast surface motion of subject S1. Upper plot: The quiver demonstrates the motion direction and amplitude, and the color encodes the motion direction, with 0° corresponding to purely horizontal motion in positive *x*‐direction, and 90° corresponding to purely vertical motion in positive *y*‐direction. Lower plot: the blue line represents the respiratory belt indications, and the red circle corresponds to the current time.

**Video S3.** The breast surface motion of subject S2. Upper plot: The quiver demonstrates the motion direction and amplitude, and the color encodes the motion direction, with 0° corresponding to purely horizontal motion in positive *x*‐direction, and 90° corresponding to purely vertical motion in positive *y*‐direction. Lower plot: the blue line represents the respiratory belt indications, and the red circle corresponds to the current time.

**Video S4.** The breast surface motion of subject S3. Upper plot: The quiver demonstrates the motion direction and amplitude, and the color encodes the motion direction, with 0° corresponding to purely horizontal motion in positive *x*‐direction, and 90° corresponding to purely vertical motion in positive *y*‐direction. Lower plot: the blue line represents the respiratory belt indications, and the red circle corresponds to the current time.

**Video S5.** The breast surface motion of subject S4. Upper plot: The quiver demonstrates the motion direction and amplitude, and the color encodes the motion direction, with 0° corresponding to purely horizontal motion in positive *x*‐direction, and 90° corresponding to purely vertical motion in positive *y*‐direction. Lower plot: the blue line represents the respiratory belt indications, and the red circle corresponds to the current time.

**Video S6.** The breast surface motion of subject S5. Upper plot: The quiver demonstrates the motion direction and amplitude, and the color encodes the motion direction, with 0° corresponding to purely horizontal motion in positive *x*‐direction, and 90° corresponding to purely vertical motion in positive *y*‐direction. Lower plot: the blue line represents the respiratory belt indications, and the red circle corresponds to the current time.

**Video S7.** The breast surface motion of subject S6. Upper plot: The quiver demonstrates the motion direction and amplitude, and the color encodes the motion direction, with 0° corresponding to purely horizontal motion in positive *x*‐direction, and 90° corresponding to purely vertical motion in positive *y*‐direction. Lower plot: the blue line represents the respiratory belt indications, and the red circle corresponds to the current time.
