# Supplementary material for: Feasibility of online non‐rigid motion correction for high‐resolution supine breast MRI
Source: Magn Reson Med. 2023 Jun 28;90(5):2130–43. doi: 10.1002/mrm.29768 (PMC10953366; doi:10.1002/mrm.29768)
Supplement: Supplementary file 1 — File S1. Background masking algorithm. [file MRM-90-2130-s003.docx]

# Segmentation algorithm for background masking

In order to remove the background artifacts, the images were roughly segmented and the background was then masked out. An example of such post-processing is demonstrated in Figure below:


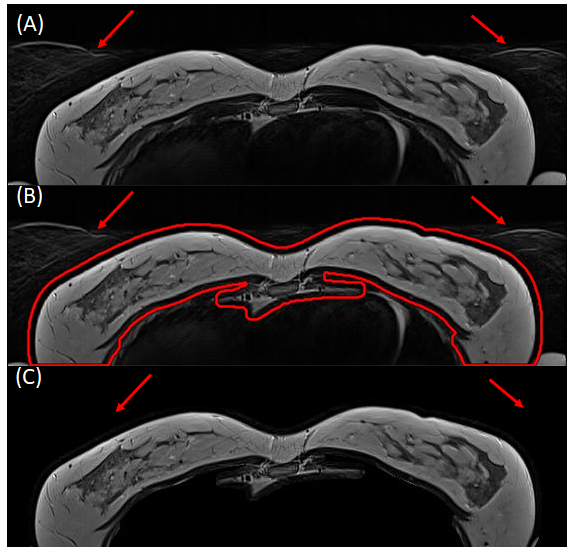


(A) A GRICS-reconstructed normalized slice before background masking. (B) Automatically selected mask (the red contour). (C) The same slice after the background masking. The red arrows show regions with residual background artifacts which do not affect diagnostics quality.

The code was implemented in MATLAB R2020a using its built-in functions. The following steps were applied:

1. Filter with a Gaussian filter (with σ=2).
2. Apply fuzzy c-means clustering (by pixel intensity) with *N_clusters_*=2.
3. Apply thresholding on the resulting probability maps with a threshold value defined for each volunteer individually. Fattier the breast is, higher threshold should be chosen.
4. Take the brighter cluster.
5. Apply morphological dilation with a disk of radius 1 (this step was necessary to fill small gaps which are present in case of a dense breast).
6. Search for connected components and select the largest component (this step removes other bright regions lying in the background).
7. Fill holes on the mask.
8. Apply dilation with a disk of radius of 7 pixels (this step ensures that all the boundaries are included to the mask for correct metrics calculation).
